# Systemic sarcoidosis presenting as a rare combination of interstitial nephritis with necrotizing vasculitis and urinary retention due to prostate involvement: a case report

**DOI:** 10.1186/s12882-023-03430-9

**Published:** 2023-12-13

**Authors:** Arata Osanami, Tomohisa Yamashita, Shintaro Sakurada, Tatsuya Sato, Yuki Kyoda, Tetsuya Shindo, Hiromi Fujita, Yayoi Ogawa, Masato Furuhashi

**Affiliations:** 1 Department of Cardiovascular, Renal and Metabolic Medicine, Sapporo Medical University School of Medicine, South-1, West-16, Chuo-Ku, Sapporo, 060-8556 Japan; 2 Department of Nephrology, Sapporo Central Hospital, Sapporo, Japan; 3 Department of Urology, Sapporo Medical University School of Medicine, Sapporo, Japan; 4 Department of Surgical Pathology, Sapporo Medical University School of Medicine, Sapporo, Japan; 5 Hokkaido Renal Pathology Center, Sapporo, Japan

**Keywords:** Sarcoidosis, Vasculitis, Interstitial nephritis, Prostate involvement, Acute kidney injury, Urinary retention

## Abstract

**Background:**

Sarcoidosis affects multiple organs and exhibits diverse clinical manifestations. Although tubulointerstitial nephritis is a known feature of renal involvement, necrotizing vasculitis is rare. Furthermore, prostate involvement with urinary retention is unusual in patients with sarcoidosis. Here, we report a case of systemic sarcoidosis with a rare combination of manifestations and different acute kidney injuries.

**Case presentation:**

A 66-year-old man developed sudden urinary retention and fever. He was diagnosed with prostatitis and admitted to our hospital. An indwelling urethral catheter was inserted, and antimicrobial therapy was initiated; however, the prostatitis was refractory. Computed tomography revealed enlarged mediastinal lymph nodes. Analysis of transbronchoscopic lymph node and prostate biopsies showed epithelioid cell granulomas, suggesting systemic sarcoidosis. During the clinical course, the serum creatinine level rapidly increased to 2.36 mg/dL without oliguria. A kidney biopsy revealed tubulointerstitial injury with moderate lymphohistiocytic infiltration and small-vessel vasculitis in the interstitium. Following oral administration of 60 mg/day prednisolone, the patient’s renal function immediately improved, and urinary retention did not recur.

**Conclusions:**

To the best of our knowledge, this is the first reported case of sarcoidosis with two unusual complications. Given its clinical course and pathology, this case is clinically valuable.

**Supplementary Information:**

The online version contains supplementary material available at 10.1186/s12882-023-03430-9.

## Background

Sarcoidosis is a systemic inflammatory disease with various clinical presentations that commonly involves the lungs, skin, lymph nodes, eyes, and other organs, including the kidneys [1, 2]. Granulomatous inflammation is the hallmark pathological feature of renal involvement in sarcoidosis. Although the condition exhibits diverse kidney manifestations, including interstitial nephritis and glomerular disease, small-vessel vasculitis is rare. Moreover, prostate involvement in sarcoidosis is unusual, with few cases of prostate-related symptoms having been reported [3]. Herein, we present an extremely rare case of sarcoidosis with acute kidney injury (AKI) due to a combination of interstitial nephritis with small-vessel vasculitis and urinary retention caused by prostate involvement.

## Case presentation

### Clinical history and initial laboratory data

A 66-year-old Japanese man with a history of hyperuricemia and bronchitis developed sudden urinary retention and fever and was diagnosed with prostatitis. Three days after onset, he received 500 mg/day levofloxacin and 8 mg/day silodosin; however, the voiding condition did not improve, and an indwelling urethral catheter was inserted. Since his symptoms persisted, the patient was admitted to the Department of Urology at Sapporo Medical University Hospital. His existing medications included febuxostat, theophylline, ambroxol hydrochloride, and long-acting β-agonist/inhaled corticosteroid combination therapy. He had no urinary symptoms related to benign prostatic hyperplasia prior to this event. On admission, the patient’s blood pressure was 139/75 mmHg, pulse rate was 85 bpm, and body temperature was 37.5 ºC. Initial laboratory tests indicated mild impairment of renal function, with an estimated glomerular filtration rate (eGFR) of 51 mL/min/1.73 m^2^ and a serum creatinine (SCr) level of 1.09 mg/dL. C-reactive protein was elevated at 14.0 mg/dL, whereas serum calcium levels remained within the normal range at 9.4 mEq/L. Notably, the level of prostate-specific antigen (PSA) was elevated at 16.3 ng/mL. Urinalysis revealed dipstick proteinuria (1 +) without pyuria or casts. Although microscopic hematuria was observed on urine sedimentation upon admission, subsequent urinalysis did not show hematuria, suggesting that the insertion of a urethral catheter may have caused temporary microscopic hematuria. Both blood and urine culture tests yielded negative results for bacterial infection. Additionally, Skin rashes were observed on his face and extremities, with a skin biopsy confirming the diagnosis of erythema nodosum. Although meropenem (1000 mg/day intravenous) was administered, his clinical status did not improve. The patient also developed misty vision and was diagnosed with uveitis by an ophthalmologist. Contrast-enhanced computed tomography (CT) revealed lymphadenopathy of the hilar, mediastinal, inguinal, and other lymph nodes. Furthermore, 18-F fluorodeoxyglucose positron emission tomography/CT showed uptake in the same lymph node, subcutaneous soft tissue and spleen and diffuse mild uptake in the kidneys (Supplemental Fig. 1) and the prostate (Supplemental Fig. 2A and B). Transbronchoscopic lymph node biopsy revealed epithelioid cell granulomas, which led to the diagnosis of systemic sarcoidosis. Although initial laboratory tests showed a mild decrease in eGFR, renal function rapidly declined during hospitalization. Following consultation with a nephrologist, laboratory tests showed renal failure, with an eGFR of 22.4 mL/min/1.73 m^2^ and an SCr level of 2.2 mg/dL. Additional urinalysis showed elevated β2-microglobulin (β2MG; 38,952 µg/L) and proteinuria (0.45 g/gCr) without hematuria. Additional laboratory tests showed elevated serum levels of soluble interleukin-2 receptor (3010 U/mL) and 1,25-dihydroxyvitamin D (85 pg/mL). Serum intact PTH levels (47.0 pg/mL) and angiotensin-converting enzyme levels (10.1 U/L) were within the normal range. Tests for myeloperoxidase anti-neutrophil cytoplasmic antibody (MPO-ANCA), proteinase 3 anti-neutrophil cytoplasmic antibody (PR3-ANCA), and anti-glomerular basement membrane antibody were all negative. The workup was negative for neoplasia; autoimmune disorders, eosinophilic granulomatosis with polyangiitis, Behçet’s disease, and cryoglobulinemic vasculitis; and infectious diseases, including fungi and *Mycobacterium tuberculosis*. Kidney and prostate biopsies were performed to confirm the histological diagnosis of systemic sarcoidosis.

**Fig. 1:**
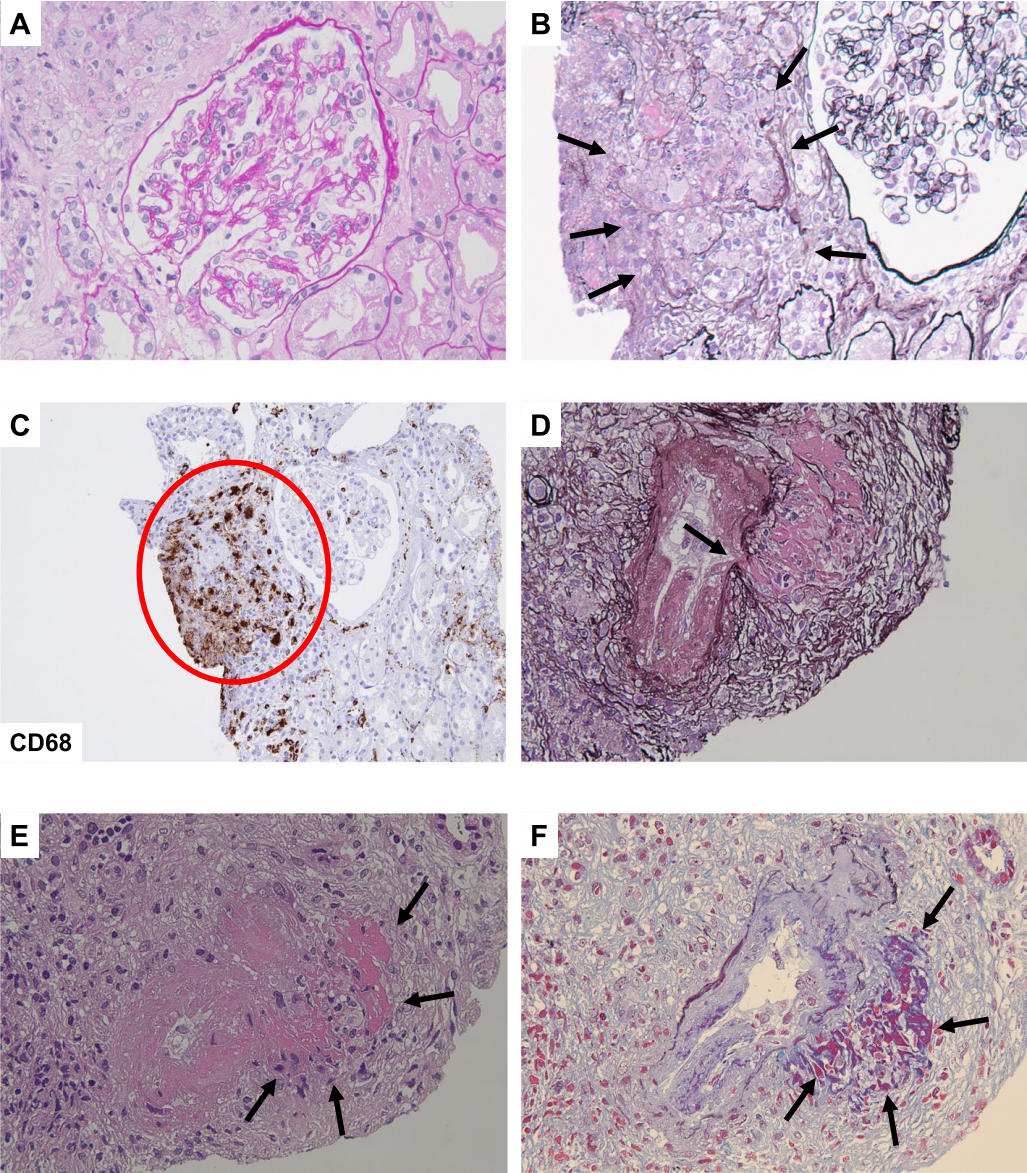
Pathological results for renal and prostate biopsy specimens. **A** No glomeruli with hypercellularity, capillary wall abnormality, or crescents were identified (renal histology of biopsy specimen, periodic acid-Schiff staining, original magnification, × 400). **B** Granulomatous inflammation in the interstitium (black arrows) (renal histology of biopsy specimen, periodic acid-methenamine-silver staining, original magnification, × 400). **C** Immunohistochemistry for CD68 reveals that many inflammatory cells are monocytes/macrophages (red circle). **D** Necrotizing vasculitis of a small artery with disruption of the elastic lamina (black arrow) (renal histology of biopsy specimen, periodic acid-methenamine-silver staining, original magnification, × 400). **E** and **F** Necrotizing vasculitis of a small artery with fibrinoid necrosis (black arrows) (renal histology of biopsy specimen, hematoxylin and eosin staining and elastica-masson staining, original magnification, × 400)

**Fig. 2:**
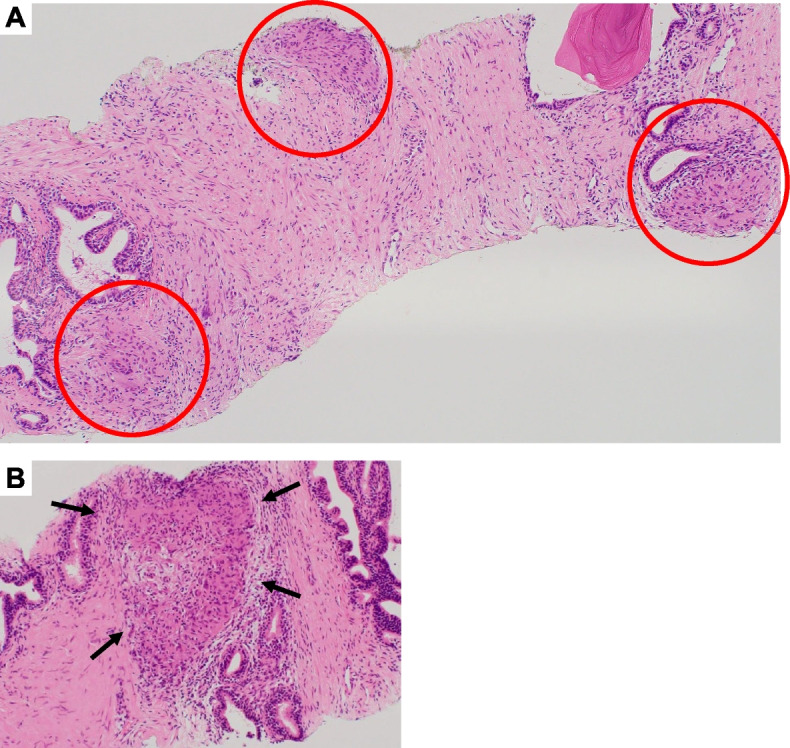
Pathological results for prostate biopsy specimens. **A** and **B** Prostate biopsy showing multiple epithelioid cell granulomas (red circles and black arrows) (prostate histology of biopsy specimen, hematoxylin and eosin staining, original magnification, × 40 and × 400)

### Pathological findings

Kidney biopsy: Light microscopy revealed the presence of 16 glomeruli, 2 of which showed global sclerosis. No glomeruli exhibited hypercellularity, capillary wall abnormalities, fibrinoid necrosis, or crescents (Fig. 1A). Tubulointerstitial injury with moderate lymphohistiocytic infiltration indicated granulomatous interstitial nephritis (Fig. 1B). Immunohistochemistry showed that the interstitial tissue was positive for the macrophage marker, CD68 (Fig. 1C). Additionally, fibrinoid necrosis and elastic lamina disruption were observed in multiple small vessels, suggesting the coexistence of necrotic vasculitis (Fig. 1D-F). Immunofluorescence microscopy revealed weakly positive immunoglobulin (Ig) A and complement component C3 depositions in the mesangial areas but negative depositions of IgG, IgM, C4, C1q, and fibrinogen. Finally, electron microscopy indicated only minor deposits in the mesangial area, without mesangial hypercellularity or changes in the capillary loop or podocytes.

Prostate biopsy: Light microscopy revealed multiple epithelioid cell granulomas (Fig. 2A and B). There was no evidence of prostate cancer or vasculitis.

### Clinical follow-up

**Fig. 3:**
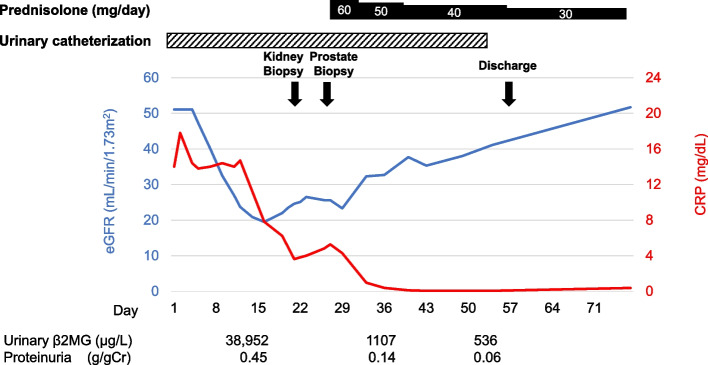
Overview of the patient’s clinical course. CRP: C-reactive protein; Urinary β2MG: urinary β2-microglobulin

The patient's clinical course following admission is shown in Fig. 3. Oral prednisolone (PSL; 60 mg/day, 1.0 mg/kg) was administered 23 days after admission. The SCr level rapidly decreased to 1.1 mg/dL 50 days after admission, and the urinary β2MG level declined to 586 µg/L. In addition, urinary retention improved after oral PSL initiation, allowing the urethral catheter to be removed. The patient was discharged 56 days after admission, and the PSL dose was gradually tapered. Seven months later, the patient remained on PSL maintenance therapy (10 mg/day), and his renal function remained stable. He had no recurrence of urinary retention, and four months after initiating treatment, his PSA level decreased to the normal range of 3.18 ng/mL.

## Discussion and conclusions

To the best of our knowledge, this is the first reported case of systemic sarcoidosis with two rare manifestations: prostate involvement and tubulointerstitial nephritis with necrotizing vasculitis. These complications resulted in different AKIs, namely post-renal AKI due to prostate involvement and renal AKI caused by granulomatous interstitial nephritis with vasculitis.

Various renal lesions have been reported in sarcoidosis [4, 5]. The most common renal manifestation is dysfunction or injury caused by abnormal calcium metabolism or granulomatous interstitial nephritis. The hallmark pathological finding of renal sarcoidosis is the presence of granulomatous inflammation. However, granulomas may be absent due to sampling error, and the presence of interstitial nephritis suggests renal sarcoidosis in the proper clinical context [6]. Renal sarcoidosis with vasculitis is unusual, with only four existing reports [7–10]. The 2012 Chapel Hill Consensus Conference classification listed sarcoid vasculitis as a differential disease associated with systemic illness [11]. Sarcoidosis has been associated with vasculitis and reportedly affects different vessel levels, ranging from small to large [12, 13]. In the previous four case reports of sarcoidosis and renal vasculitis, the administration of glucocorticoids resulted in favorable renal outcomes. It remains possible that other types of vasculitis were incidentally complicated by sarcoidosis, and weakly positive IgA and complement component C3 depositions in the mesangial areas might suggest IgA nephropathy as an underlying disease. A previous report suggested several glomerular diseases, including IgA nephropathy, are associated with sarcoidosis [14]. However, in this case, both MPO-ANCA and PR3-ANCA were negative, and the kidney biopsy showed no mesangial proliferation or hypercellularity, including crescent formation or tuft necrosis in the glomeruli. In addition, urinary findings were negative for hematuria. These results are not consistent with ANCA-associated vasculitis or activated IgA nephropathy.

The involvement of the prostate in sarcoidosis is also rare; only a few case reports with a small number of case series have been published [3, 15, 16]. Most of these patients were asymptomatic, and the diagnosis was determined by biopsy, resection of the prostate, or autopsy. Additionally, a few individuals complained of high frequency and hesitancy of micturition [3]. The mechanism of urinary retention in this case is unclear; however, the presence of multiple granulomas or severe inflammation, which was not sampled in the prostate biopsy, could contribute to bladder outlet obstruction. In addition, according to previous reports, neurosarcoidosis can cause bladder dysfunction [17, 18]. Although he did not complain of obvious neurologic symptoms, urinary retention might be attributed to nervous system involvement in this patient. Although prostate involvement in sarcoidosis might be underreported, we believe that this manifestation should be included in the differential diagnosis for patients presenting with urinary symptoms, including frequent urination or urinary retention.

Our patient showed rapid recovery of renal function and improvement of urinary retention after treatment with PSL, suggesting that renal and prostatic sarcoidosis respond well to steroid therapy. To determine the optimal treatment strategy for a given case, we emphasize the importance of histopathological analyses for the correct diagnosis of each lesion in sarcoidosis and its various clinical manifestations.

In summary, we describe a case of sarcoidosis presenting with interstitial nephritis with small-vessel vasculitis and urinary retention due to prostate involvement, which caused renal AKI and post-renal AKI, respectively. Further case reports and research studies are required to elucidate the pathogenesis and optimal management of these rare manifestations in sarcoidosis.

### Supplementary Information

**Additional file 1: Supplementary Figure S1.** FDG-PET/CT findings in the patient’s kidneys. FDG-PET/CT showing mild uptake in the kidneys (red arrows). **Supplementary Figure S2.** FDG-PET/CT findings in the patient’s prostate. (A) FDG-PET/CT showing mild uptake in the prostate (red arrows). (B) Corresponding CT image at the same level as Figure S2A, with a red arrow showing the inserted urethral catheter.

## Data Availability

The datasets used and/or analyzed during the current study are available from the corresponding author upon reasonable request.

## Abbreviations

AKI: Acute kidney injury
β2MG: β2-Microglobulin
CT: Computed tomography
Egfr: Estimated glomerular filtration rate
Ig: Immunoglobulin
MPO-ANCA: Myeloperoxidase anti-neutrophil cytoplasmic antibody
PR3-ANCA: Proteinase 3 anti-neutrophil cytoplasmic antibody
PSL: Prednisolone
SCr: Serum creatinine
