# Supplementary figures and images for: Systemic sarcoidosis presenting as a rare combination of interstitial nephritis with necrotizing vasculitis and urinary retention due to prostate involvement: a case report
Source: BMC Nephrol. 2023 Dec 13;24:370. doi: 10.1186/s12882-023-03430-9 (PMC10720170; doi:10.1186/s12882-023-03430-9)

## Slide 1
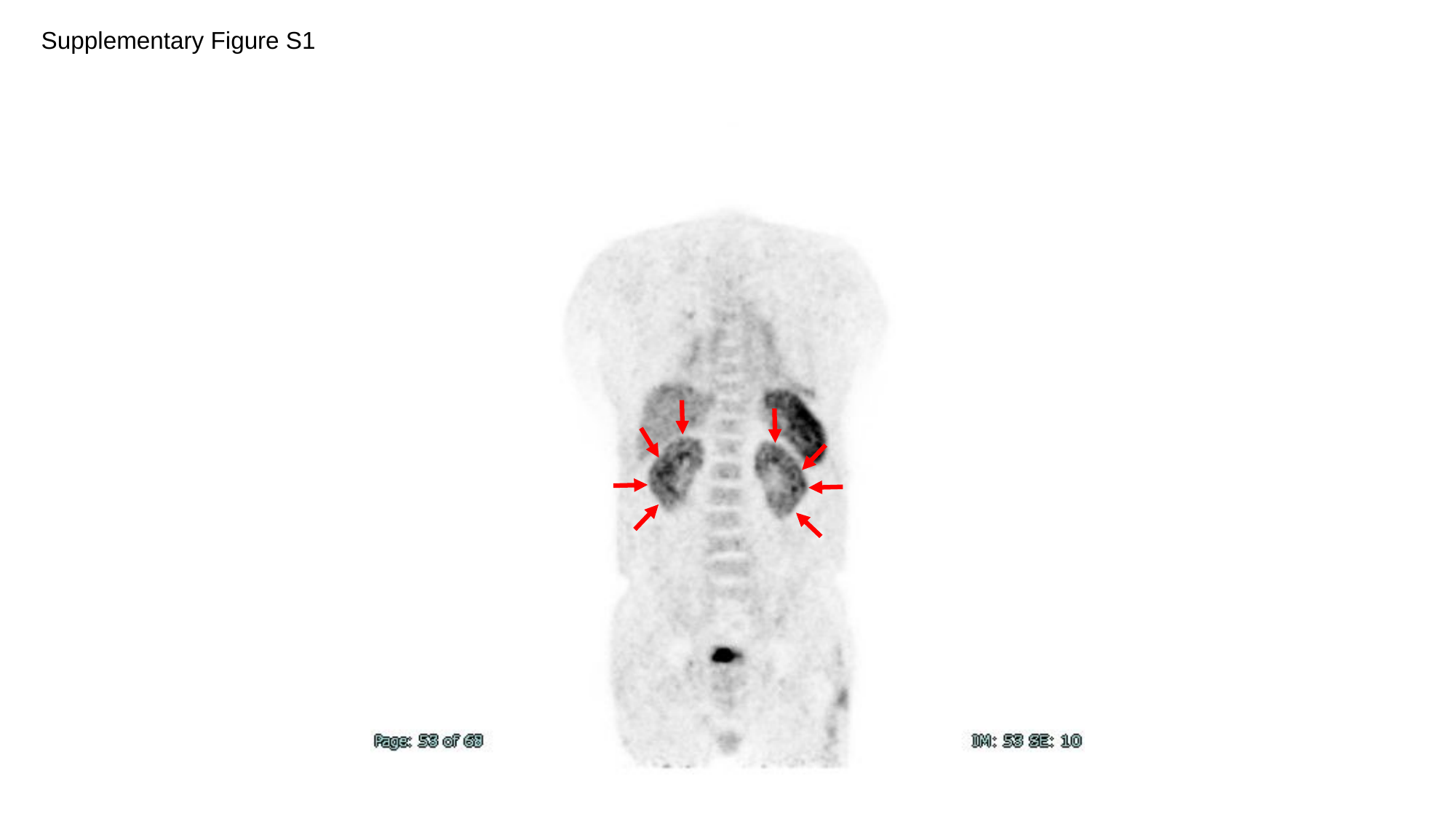

Supplementary Figure S1

Supplement: Supplementary file 1 — Additional file 1: Supplementary Figure S1. FDG-PET/CT findings in the patient’s kidneys. FDG-PET/CT showing mild uptake in the kidneys (red arrows). Supplementary Figure S2. FDG-PET/CT findings in the patient’s prostate. (A) FDG-PET/CT showing mild uptake in the prostate (red arrows). (B) Corresponding CT image at the same level as Figure S2A, with a red arrow showing the inserted urethral catheter. [file 12882_2023_3430_MOESM1_ESM.zip › 12882_2023_3430_MOESM1_ESM.pptx/Supplementary Figure S1_ESM.pptx]

## Slide 1
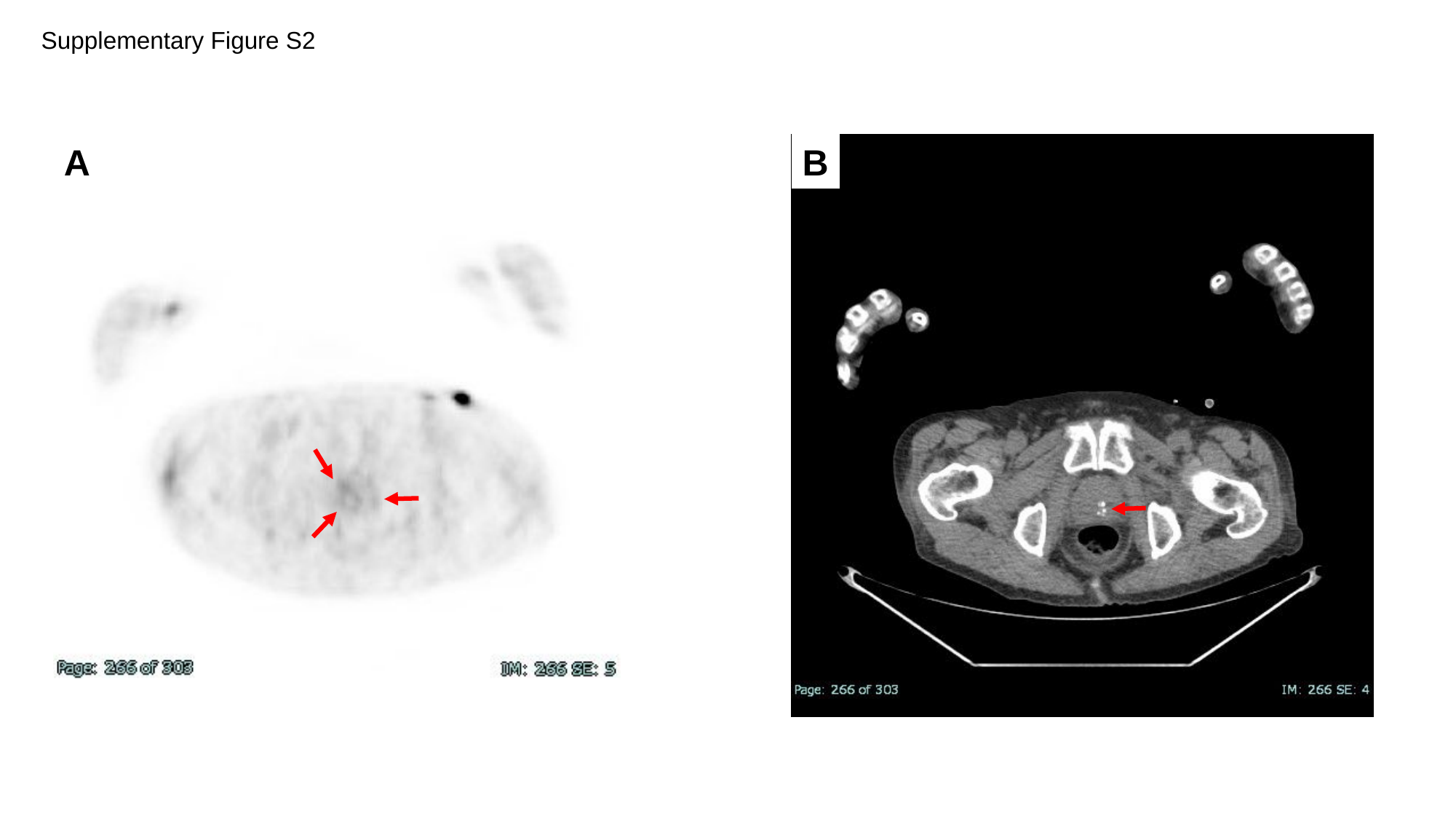

Supplementary Figure S2
A
B

Supplement: Supplementary file 1 — Additional file 1: Supplementary Figure S1. FDG-PET/CT findings in the patient’s kidneys. FDG-PET/CT showing mild uptake in the kidneys (red arrows). Supplementary Figure S2. FDG-PET/CT findings in the patient’s prostate. (A) FDG-PET/CT showing mild uptake in the prostate (red arrows). (B) Corresponding CT image at the same level as Figure S2A, with a red arrow showing the inserted urethral catheter. [file 12882_2023_3430_MOESM1_ESM.zip › 12882_2023_3430_MOESM1_ESM.pptx/Supplementary Figure S2_ESM.pptx]
